# To Accelerate or Not? Decision Making for Gifted Students: Insights from a Tri-Case Study on Cognitive-Achievement Alignment and Program Fit

**DOI:** 10.3390/bs16071230

**Published:** 2026-07-20

**Authors:** Yusra Zaki Aboud

**Affiliations:** The National Centre of Giftedness and Creativity, King Faisal University, Al-Ahsa 31982, Saudi Arabia; yozaki@kfu.edu.sa

**Keywords:** acceleration, gifted education, cognitive profile, case study, CogAT, psychosocial assessment, Saudi Arabia

## Abstract

Academic acceleration decisions for gifted students require a shift from single-scale, grade-based approaches toward integrated, multi-domain assessments that consider cognitive abilities, academic performance, and psychosocial readiness together. This mixed-methods case study examines three Saudi students who experienced academic acceleration and who present distinct cognitive–academic profiles—designated as “Outstanding” (O.F.), “Inconsistent” (O.L.), and “Inconsistent and Regressing” (O.M.)—to explore how the congruence among cognitive abilities (measured by CogAT), academic performance (evaluated through predictive descriptive modeling), and psychosocial readiness (assessed by the modified PDAS) influences acceleration decision making. Data were collected from cognitive tests, academic records, PDAS scores, and semi-structured interviews with students, teachers, and parents. Findings indicate that strong convergence across the three domains was associated with successful whole-grade acceleration, whereas domain-specific mismatch and significant weakness in fluid reasoning were associated with academic decline, psychological distress, and social difficulties. Composite scores masked substantial variations within these asymmetric patterns, leading to potentially inappropriate classifications. Although parental support varied across cases and appeared associated with student outcomes, a causal relationship could not be established within this exploratory design. The study concludes that acceleration policies should move beyond general averages toward individualized, profile-based assessments that consider subject-specific acceleration and psychosocial support when needed. All findings are preliminary observations that require replication with larger samples and longitudinal designs before definitive conclusions can be drawn.

## 1. Introduction

Academic acceleration—allowing students to progress through educational content at a faster pace or earlier than usual—has long been a controversial topic in gifted education. Proponents argue that it addresses the needs of exceptionally gifted students by providing challenging material, thereby reducing boredom, disengagement, and underachievement (Assouline et al., 2015; Steenbergen-Hu et al., 2016). Critics, however, raise concerns about social isolation, psychological pressure, and placing students in environments for which they may not be emotionally or academically prepared (Gulcin, 2021). Therefore, acceleration is not a universal solution, but rather one tool whose effectiveness depends on the context, the learner’s profile, and the support systems available.

Despite well-documented positive outcomes for appropriately selected students—including academic gains and stable or improved psychosocial well-being (Miravete, 2022; Bernstein et al., 2021)—implementing acceleration in emerging education systems like Saudi Arabia faces significant obstacles. These include the lack of standardized national procedures (Bin Yousef, 2021), a shortage of trained professionals (Aljomaih, 2023), resistance from teachers and parents (Almhawes, 2024), limited preparation time (Al-Ablan, 2021), and the risk of excessive academic pressure (Alzahrani & Youssef, 2023).

The fundamental problem is that acceleration decisions in Saudi Arabia currently rely on standardized selection criteria—primarily composite scores and overall academic averages—without considering students’ individual cognitive, academic, or psychological profiles. This approach assumes that a student excelling in all subjects automatically qualifies for full-grade acceleration. However, research indicates that acceleration is not a one-size-fits-all solution (Al-Qahtani, 2024). A student with a balanced (“equivalent”) cognitive profile may thrive, while one with an “unequal” profile (exceptional strengths alongside significant weaknesses) may be disadvantaged. Composite scores are calculated as averages across domains, masking critical discrepancies (such as deficits in quantitative or nonverbal reasoning) and potentially leading to inappropriate decisions and psychological and social distress.

To date, very limited scientific evaluation of acceleration outcomes in Saudi Arabia has been conducted. Available reports have focused on the quality of services, rather than on cognitive, academic, or psychosocial outcomes (Alarfaj & Al-Omair, 2020; Alfaiz et al., 2022; Almhawes, 2024; Y. Aboud, 2021; Bin Yousef, 2021). No known Saudi study has systematically compared students with different cognitive and academic profiles using a comprehensive, multi-perspective methodology.

Therefore, this study adopts a qualitative–quantitative, multi-case, and multi-perspective design—a novel approach in the Saudi context. It examines how individual acceleration decisions can be based on the alignment of three elements: cognitive profile (CogAT test), academic trajectory (predictive modeling), and psychosocial readiness (adapted PDAS). By comparing the perspectives of accelerated students, their teachers, and their parents, the study goes beyond assessing service quality to examining cognitive, academic, and psychosocial outcomes.

Based on this gap in the research, we analyze three case studies of individual Saudi students who have been accelerated and who each have different cognitive, academic and psychosocial profiles. The purpose of this research is to explore whether an integrated case study model with a mixed methodological approach, integrating cognitive, academic, and psychosocial data, can generate research questions and provide preliminary insights to guide individual acceleration decisions for gifted students. By examining the congruence or incongruence of these dimensions, this study offers preliminary insights to help guide future research, which can then in turn provide evidence to inform acceleration policies and practices in Saudi Arabia and beyond.

## 2. Literature

### 2.1. Academic Acceleration

Academic acceleration—the practice of allowing students to move through educational content at a pace or age earlier than typical—is one of the most extensively researched and empirically validated interventions for students whose learning outpaces that of their age peers (Steenbergen-Hu et al., 2016; Miravete, 2022). This study focuses specifically on whole-grade acceleration (grade skipping), which involves placing a student in a grade level above their age peers (Assouline et al., 2015).

Gifted students learn quickly, comprehend complex topics, and possess a unique ability to connect ideas, requiring differentiated educational programs that foster linguistic and logical skills, creativity, and innovative thinking (Gulcin, 2021). However, their needs are often overlooked due to lack of resources (Y. Z. Aboud, 2026), uninspiring traditional learning environments (Alarfaj & Al-Omair, 2020), and unsuitable curricula (Schuur et al., 2020), leading to boredom, academic decline, loss of passion for learning, and behavioral issues.

Academic acceleration addresses these challenges by providing content and cognitive challenges that match gifted students’ advanced levels (Ogurlu, 2021; Bernstein et al., 2021 Miravete, 2022), protecting them from intellectual stagnation, unlocking their potential, and enhancing motivation and self-confidence (Steenbergen-Hu & Moon, 2011). It also improves psychological well-being and happiness when abilities are recognized and valued (Schuur et al., 2020; Yuen et al., 2026). Acceleration serves as an acknowledgment of individual differences and a commitment to educational flexibility, empowering gifted students to reach their full potential (Al-Qahtani, 2024). Technology has further increased flexibility in content delivery and teaching methods, making it an ideal option for accelerating gifted learners (Y. Aboud, 2025).

### 2.2. Evidence and Implementation Challenges

A substantial body of research supports acceleration’s effectiveness for appropriately selected students (Assouline et al., 2015; Steenbergen-Hu et al., 2016). However, implementation remains debated among parents, educators, and researchers (Alarfaj & Al-Omair, 2020).

#### 2.2.1. Social and Emotional Outcomes

Concerns that accelerated students may experience social isolation, psychological distress, or difficulty forming peer relationships (Gulcin, 2021) are consistently challenged by research. A meta-analysis by Steenbergen-Hu et al. (2016) found that accelerated children outperform non-accelerated peers academically while exhibiting equivalent or better social and emotional adjustment. A 35-year longitudinal study by Bernstein et al. (2021) demonstrated that concerns about socio-emotional effects are unfounded. When thoughtfully implemented, acceleration maintains or enhances dimensions of well-being, including social interactions, self-concept, and emotional adjustment (Assouline et al., 2015; Schuur et al., 2020). However, positive outcomes are not automatic and may depend on alignment between student profile and intervention (Cross et al., 2018; Gulcin, 2021).

#### 2.2.2. Implementation Challenges

Implementation faces significant barriers. According to the National Association for Gifted Children (2022), only approximately 30% of U.S. schools implement acceleration regularly. Misconceptions among teachers and administrators, often stemming from insufficient professional preparation, contribute to underutilization. In Saudi Arabia, Y. Aboud (2023) reported that only 16% of teachers have completed a course in gifted education. Research indicates that when planned correctly, grade skipping does not lead to social or emotional problems (Assouline et al., 2015; Yuen et al., 2026), with success depending on aligning acceleration with the student’s personality and individual needs, including self-confidence, motivation, and a supportive environment (Y. Aboud, 2025).

#### 2.2.3. Curricular Limitations

Acceleration often fails to provide a truly differentiated curriculum for gifted learners. Accelerated students frequently receive instruction designed for older, average students rather than adapted content, missing opportunities for qualitatively different educational experiences that gifted learners require to fully develop their potential.

The literature reviewed above demonstrates that while acceleration is a well-supported intervention, its success depends on factors that extend beyond simple composite measures. However, no single theoretical framework adequately captures the multidimensional nature of acceleration decisions. To address this complexity, the following section synthesizes four complementary theoretical models that collectively inform the study’s integrated assessment approach. These models—spanning cognitive differentiation (CHC theory), profile patterns (Equivalent/Uneven framework), environmental fit (Person–Environment Fit theory), and ecological systems (Bronfenbrenner’s model)—provide the conceptual tools necessary to examine how alignment across cognitive, academic, and psychosocial domains may influence acceleration outcomes. Each model is first presented individually, then synthesized in Section 3.6 to demonstrate their complementary application to the present study.

## 3. Theoretical Models

### 3.1. The Cattell–Horn–Carroll (CHC) Theory of Cognitive Abilities

The Cattell–Horn–Carroll (CHC) theory is the most comprehensive and empirically validated contemporary framework for understanding the structure of human cognitive abilities (McGrew, 2009; Schneider & McGrew, 2018). Rather than reducing intelligence to a single, global “g-factor,” the theory posits a hierarchical, multidimensional architecture comprising three strata: Stratum I encompasses narrow, specific abilities such as lexical knowledge or mathematical achievement; Stratum II includes broad abilities such as fluid intelligence (*Gf*), crystallized intelligence (*Gc*), visual processing (*Gv*), and quantitative knowledge (*Gq*); and Stratum III represents general intellectual functioning.

The theoretical distinction between fluid intelligence (*Gf*) and crystallized intelligence (*Gc*) forms a foundational pillar of this study. *Gf* represents the capacity to reason, solve novel problems, identify patterns, and draw inferences independent of prior learning or cultural exposure—it is the engine of analytical thinking. In contrast, *Gc* represents the accumulation of knowledge, vocabulary, and skills acquired through education and cultural experience Cattell, 1987, as cited in (Thorndike & Hagen, 1996). A critical implication of this is that an individual’s cognitive profile is inherently multidimensional; a student can simultaneously possess exceptional *Gc* (e.g., advanced verbal reasoning) while exhibiting below-average *Gf* (e.g., difficulties with novel, non-verbal problem-solving). Consequently, a single composite score necessarily masks these differential strengths and weaknesses, obscuring the very information most relevant to educational placement.

The relevance of CHC theory to academic acceleration decisions lies in its challenge to the assumption that a student is uniformly prepared to engage with all content at a higher grade level, by revealing that students often present with significantly “uneven” cognitive architectures. A student with high *Gc* but low *Gf* is likely to succeed in fact-based, language-heavy subjects such as biology, computer science, and literature, but struggle critically in mathematics and physics—disciplines that demand novel problem-solving, spatial reasoning, and abstract pattern recognition. Similarly, a deficit in *Gq* (quantitative knowledge) predicts difficulty with the computational and numerical demands of an accelerated STEM curriculum. Accordingly, the theory provides the essential justification for moving beyond composite scores in acceleration decisions, as it posits that cognitive profile—not general ability alone—determines the appropriateness of specific educational interventions. A student with an “Equivalent High” profile, uniformly high across *Gf*, *Gc*, and *Gq*, is theoretically well-suited to whole-grade acceleration, whereas a student with an “Uneven” profile, such as high *Gc* with low *Gf* and *Gq*, requires targeted, domain-specific supports or subject-based acceleration rather than a blanket grade-skip.

In this study, CHC theory functioned as the primary cognitive lens governing sampling, instrumentation, and data interpretation as follows. Regarding instrumentation, the Cognitive Abilities Test (CogAT) was selected not merely as a validated psychometric tool, but specifically because its three batteries directly operationalize CHC’s broad-ability constructs: the Verbal battery measures *Gc*, the Quantitative battery measures *Gq*, and the Nonverbal battery measures *Gf*. This theoretical alignment ensures that the test captures the multidimensionality that CHC theory deems essential, rather than collapsing abilities into a single score. Regarding case selection, the theory directly informed the purposive sampling criteria, with the three cases explicitly selected to reflect distinct CHC-derived profiles—the Equivalent High profile, the Uneven profile, and the Declining Uneven profile—allowing the study to test whether CHC-predicted patterns of academic strength and weakness would manifest in real-world accelerated settings. Regarding data interpretation, CHC theory provided the explanatory mechanism linking cognitive scores to academic outcomes throughout the Section 6 and Section 7. In this way, all interpretive claims regarding cognitive–academic alignment are explicitly grounded in CHC’s dimensional framework, ensuring that the study’s conclusions are theory-driven rather than merely descriptive

### 3.2. The Equivalent vs. Uneven Cognitive Profile Framework

The Equivalent vs. Uneven Cognitive Profile Framework offers a method for classifying students based on their pattern of performance across different cognitive domains rather than relying on a single composite score, operating on the fundamental premise that students may exhibit varying patterns of cognitive strengths and weaknesses across domains (Lohman & Lakin, 2017). Equivalent profiles, characterized by consistently high scores with minimal variation, are generally considered to indicate that a student may be well-suited for full-grade acceleration, whereas uneven profiles showing significant variation across domains—such as exceptional strengths alongside average or below-average performance—may suggest that targeted acceleration or enrichment could be more appropriate options.

It is important to distinguish this framework from the “Patterns of Strengths and Weaknesses” (PSW) frameworks used in learning disabilities identification contexts (Miciak et al., 2014), such as the Consistency/Discrepancy Method and the Cross-Battery Assessment method, which are primarily designed to identify students with learning disabilities by examining specific discrepancies between cognitive abilities and academic achievement and have been shown to suffer from low agreement rates and limited validity evidence. In contrast, the Equivalent/Uneven profile framework employed in this study differs in purpose, as it serves not as a diagnostic tool for learning disabilities but as a framework for educational decision making regarding academic acceleration for gifted students; it differs in theoretical basis, being derived from CHC theory and focusing on patterns of broad abilities rather than on fine-grained discrepancies between abilities and achievement; and it differs in application, being used to guide placement decisions such as full acceleration, subject-specific acceleration, or enrichment rather than to identify disabilities.

This framework played a pivotal role in shaping the study at three levels. First, it served as the direct basis for the purposive sampling strategy, with the three cases explicitly selected to represent the three patterns identified through preliminary analysis of academic records of forty-five accelerated students: the Equivalent High profile characterized by minimal variance across cognitive and academic domains, the Uneven profile showing significant variance between verbal and quantitative or nonverbal abilities, and the Declining Uneven profile marked by notable weakness in fluid reasoning with a sharp decline in quantitative subjects after acceleration. This provided the theoretical justification for selecting cases representing the full range of observed variance, enabling meaningful comparative analysis. Second, the framework specified that significant variance is considered meaningful when the standard deviation exceeds 1.5 or the range exceeds twenty percentile points, and these descriptive thresholds based on expert consensus and practical tradition were used to classify cases and guide interpretation of CogAT results, as exemplified by Student S.L. whose standard deviation across the three batteries was 28.2, far exceeding the 1.5 threshold and confirming her classification as an uneven profile. Third, the framework provided an interpretive lens for connecting cognitive patterns to academic outcomes.

The Equivalent/Uneven profile framework works alongside CHC theory by providing a mechanism for classifying and interpreting the patterns that CHC theory identifies; while CHC theory specifies the cognitive dimensions to be measured, the framework provides a way to organize and interpret variation across these dimensions. This framework also integrates with Person–Environment Fit theory by assuming that uneven profiles may signal potential misfit with the demands of the accelerated classroom, warranting consideration of alternatives such as subject-specific acceleration or additional support.

### 3.3. Person–Environment Fit Theory

The Person–Environment Fit theory, rooted in occupational and educational psychology (Hanna et al., 2021), posits that optimal outcomes are more likely when there is a good fit between an individual’s characteristics and the demands of their environment. When applied to academic acceleration, this theory suggests that a good fit between a student’s cognitive abilities, academic skills, emotional maturity, and social competencies on the one hand, and the expectations and dynamics of the accelerated classroom on the other, may increase the chances of successful adjustment. However, the theory does not identify which dimensions of fit are most important, nor does it fully consider the possibility of modifying the environment to better support the student. Furthermore, perceived conformity—the subjective sense of belonging—may differ from objective conformity, and these discrepancies are not yet well understood. Therefore, the theory is used here as an interpretive lens rather than a predictive engine, meaning that in practice, using person–environment alignment theory as an interpretive lens involves asking where objective alignment between the student’s abilities and maturity and the demands of the class aligns or fails to align with the student’s sense of belonging. Since the theory does not prioritize dimensions of alignment or address environmental modification, it has been used in conjunction with congruence theory, where the latter addresses subjective belonging and the discrepancies between the perceived and the objective, while the former organizes the analysis of objective alignment. The two theories are not used in isolation; congruence theory serves as the interpretive lens, and person–environment alignment theory provides the core conceptual vocabulary.

This theory directly shaped the semi-structured interview questions for students, teachers, and parents by structuring protocols to probe specific dimensions of fit rather than asking general questions about the acceleration experience. These dimensions included pace fit as reflected in whether the student felt the class moved at a comfortable speed, social fit as reflected in whether the student felt they belonged with their classmates, academic challenge fit as reflected in whether assignments were appropriately challenging or overwhelming, and expectation fit as reflected in whether the student felt able to meet the teacher’s expectations. This theory-driven questioning ensured that interviews captured the specific misfit patterns that the theory predicts would lead to negative outcomes. The thematic analysis of interview transcripts was guided by Person–Environment Fit theory, with responses coded for evidence of fit or misfit across three environmental dimensions: cognitive and pace fit regarding whether the student felt the accelerated pace matched their learning speed, social fit regarding whether the student felt socially integrated and accepted, and expectation fit regarding whether the student felt able to meet academic and social expectations.

Person–Environment Fit theory also provided the explanatory mechanism for why students with similar cognitive profiles might have different acceleration experiences, suggesting that a student may have adequate cognitive abilities but still experience difficulties if there is misfit in other dimensions such as social, emotional, or expectations. Furthermore, while the theory traditionally focuses on selecting students who fit the environment, this study extended the theory to consider whether the environment could be modified to better fit the student. The recommendation for subject-specific acceleration for Student S.L. and the recommendation for academic intervention and psychosocial support for Student T.M. represent efforts to modify the environment rather than simply labeling the student as a “poor fit,” acknowledging that fit is bidirectional and that educational systems bear responsibility for adapting to student needs.

### 3.4. Psychosocial Support Ecosystem Model (Bronfenbrenner)

The Bronfenbrenner model constitutes a cornerstone of this study’s theoretical foundation, providing a socio-ecological lens through which the accelerated student’s environment is understood as comprising multiple, interconnected layers of influence. This framework serves a dual purpose: it organizes our understanding of the sources of support and pressure that may shape acceleration outcomes, while simultaneously justifying the multi-perspective data collection approach that lies at the heart of this research. Bronfenbrenner, 1979, as cited in (El Zaatari & Maalouf, 2022) ecological theory distinguishes four interrelated systems operating at different levels of proximity to the student. The microsystem encompasses the immediate environment where the student directly interacts with peers, teachers, and family—the level at which acceleration is most directly experienced. The mesosystem captures the interactions between these microsystem components, such as how teacher expectations influence parental involvement or how peer relationships affect family dynamics. The exosystem comprises the broader institutional and political contexts that indirectly affect the student, including school acceleration policies, teacher training programs, and resource allocation decisions. Finally, the macrosystem encompasses cultural attitudes, societal values, and prevailing beliefs about gifted education that shape the broader context within which all other systems operate.

This ecological framework directly shaped the study’s design in several fundamental ways. The Psychosocial and Dispositional Assessment Scale (PDAS) was specifically constructed to capture the microsystem level within this model, with its four dimensions—emotional development, peer relationships, student attitude, and parental attitude and support—directly operationalizing key microsystem components. Emotional development captures the student’s internal psychological microsystem; peer relationships reflect the social microsystem; student attitude represents the motivational microsystem; and parental attitude embodies the family microsystem. This multidimensional approach ensures that the PDAS provides a comprehensive assessment of the immediate environment rather than a single, narrow measure. Beyond the PDAS, Bronfenbrenner’s model provided the theoretical justification for the study’s multi-perspective design, with semi-structured interviews involving students, teachers, parents, and school counselors designed to gather information across all four ecological levels. These interviews captured direct experiences within the microsystem, explored mesosystem interactions such as how teacher comments about uneven performance influenced parental anxiety and pressure, examined exosystem factors including school policies and the Saudi National Acceleration Program’s reliance on composite scores, and investigated macrosystem influences such as cultural attitudes toward gifted education in Saudi Arabia.

When applied to data interpretation, Bronfenbrenner’s model enabled the identification of multi-level influences on acceleration outcomes that would otherwise remain invisible.

Perhaps most importantly, Bronfenbrenner’s model provides a roadmap for intervention by identifying precisely where and at which system level action is most needed. This guided the study’s recommendations toward targeted, multi-level strategies rather than generic advice. Exosystem interventions called for school-level policy changes to allow subject-specific acceleration as an alternative to whole-grade acceleration for students with uneven profiles. Finally, macrosystem interventions pointed toward cultural awareness and professional development initiatives to shift attitudes away from composite-score-based decisions toward profile-based assessments. By locating interventions within these specific system levels, Bronfenbrenner’s model transformed the study’s recommendations from general advice into precise, multi-level strategies that address not only the individual student but the broader ecological context in which acceleration decisions are made, ensuring that support is both comprehensive and appropriately targeted.

### 3.5. Integrated Acceleration Decision-Making Model

The current study proposes an integrated model that attempts to synthesize three broad areas: cognitive assessment (based on CHC theory to examine abilities specific to each area), analysis of academic achievement (using predictive linear modeling to compare actual performance with expectations), and assessment of psychosocial readiness (via the PDAS adapted from the Iowa Acceleration Scale; Assouline et al., 2009).

The central, still exploratory, hypothesis is that the success of the acceleration decision may depend on the degree of convergence among these three domains. However, the model has not yet been empirically validated in large-scale or multicultural contexts; therefore, its usefulness should be viewed as hypothesis-generating rather than prescriptive. The weight given to each domain is likely to vary according to individual student characteristics and contextual factors, and ongoing monitoring is essential to assess whether convergence persists over time.

### 3.6. Synthesis for the Present Study

The four theoretical models function as an integrated analytical framework rather than isolated perspectives, with each model addressing a distinct yet complementary question about acceleration decisions. Bronfenbrenner’s ecological model provides the overarching map of environmental levels—microsystem, mesosystem, exosystem, and macrosystem—while Person–Environment Fit theory offers the compass for assessing alignment or misalignment within each level. These two models work in tandem: Bronfenbrenner determines which stakeholders should be interviewed across different ecological levels, while fit theory shapes the specific questions asked about belonging, challenge, and expectations. The cognitive models—CHC theory and the Equivalent/Uneven profile framework—operate at the individual level to describe and classify the student’s cognitive architecture, with CHC specifying the dimensions to be measured and the profile framework providing criteria for interpreting variation across those dimensions.

This theoretical integration directly informs every methodological choice. The study adopts a quasi-positivist pragmatism (Creswell & Plano Clark, 2018) wherein quantitative instruments measure objective patterns and qualitative interviews capture subjective interpretations, with both approaches derived from the theoretical models. CHC theory guides the selection and interpretation of the CogAT test; the Equivalent/Uneven framework establishes the case selection criteria; Person–Environment Fit theory structures the semi-structured interview protocols; and Bronfenbrenner’s model underpins both the multi-perspective design and the dimensional structure of the PDAS. During analysis, theoretically oriented thematic coding examines compatibility and inequality both across cognitive, academic, psychological, and social domains and within each ecological level, with these patterns ultimately integrated into holistic case narratives. This integrated approach transforms the models from background references into active, practical tools that inform sampling, instrumentation, data collection, analysis, and interpretation at every stage, ensuring that the study’s conclusions are consistently theory-driven rather than merely descriptive.

## 4. Materials and Methods

### 4.1. Study Design: A Mixed-Methods Instrumental Case Study

This study employs a mixed-methods design, specifically an instrumental case study (Creswell & Plano Clark, 2018; Yin, 2018). By “mixed-methods,” we mean the simultaneous collection, analysis, and integration of both quantitative and qualitative data within a single study. Specifically, quantitative data (CogAT scores, academic records, and PDASs) are used to measure cognitive abilities, academic performance, and psychosocial readiness in a standardized, numerically quantifiable manner. Qualitative data (semi-structured interviews with students, teachers, and parents) are used to explore participants’ perspectives, experiences, and interpretations in depth. The researcher used Dudovskiy’s (2018) qualitative data analysis approach to examine the details of the data to track any noteworthy recurring themes or patterns that were consistent with the study’s objective. Using Nvivo version 20 coding, the study data were analyzed. Both types of data are collected at the same stage and analyzed independently (descriptive statistics and predictive modeling for quantitative data; thematic analysis for qualitative data). They are then integrated during the interpretation phase to compare and contrast the evidence for each case and to synthesize the contrasting findings. This integration enables triangulation—the cross-validation of results from different sources—and provides a more complete and comprehensive understanding than either type of data could offer on its own.

In instrumental case studies, cases are not examined for their intrinsic uniqueness, but rather as means to provide insight into a broader issue or to enhance theoretical understanding. Here, the three cases serve as instrumental tools for understanding the broader phenomenon of acceleration decision making and for illustrating the application of an integrated assessment model. This design is particularly well-suited for exploring complex real-world phenomena within their natural context, especially when the boundaries between the phenomenon and its context are not clearly defined (Yin, 2018).

The instrumental case study design aligns directly with the research objectives, which seek not only to describe individual experiences but also to derive transferable insights into how cognitive, academic, psychological, and social factors may interact to influence acceleration outcomes. By examining three cases representing distinct cognitive profiles, this design allows for a comparative analysis of the cases to identify patterns of alignment that contribute to the success or failure of acceleration.

The choice of a mixed-methods approach is justified by three reasons: First, academic acceleration is multidimensional by nature, encompassing cognitive, academic, and psychosocial domains that quantitative measures alone cannot fully capture. Second, the study seeks to understand not only what outcomes occur, but also why and how these outcomes emerge from the perspectives of multiple stakeholders. Third, triangulating quantitative assessment data with qualitative interview data allows for cross-validation of results and provides a more complete picture of each case.

This approach is characterized by the simultaneous collection of quantitative data (CogAT, academic records, PDAS) and qualitative data (semi-structured interviews). Independent analysis of each data type and integration during interpretation allow for a comprehensive assessment of the alignment between cognitive, academic, and psychosocial factors.

Participants were selected through purposive sampling to ensure variability in the phenomenon of interest—that is, the degree of alignment between cognitive ability, academic performance, and program suitability. The sample for each case included: (a) the accelerated student, (b) the student’s core subject teachers, and (c) the student’s parents (or one of them). This multi-perspective approach enabled the triangulation of data sources and a holistic understanding of each case.

### 4.2. Sample

The study sample consisted of three gifted high school students in the Al-Ahsa region of Saudi Arabia who were academically accelerated. They were purposefully selected to represent different patterns of cognitive and academic performance. This selection allowed for a comprehensive comparative analysis of the suitability of the academic acceleration decision for each individual case.

The three profile (pattern) definitions—”highly equivalent,” “unequal,” and “unequally decreasing”—were developed by the researcher based on a preliminary analysis of the academic records of accelerated students in the Al-Ahsa region. Over three semesters, the researcher examined the academic trajectories of 45 accelerated students. From this analysis, three distinct patterns emerged: (a) consistently high performance in all subjects, (b) high performance in some subjects coupled with persistent weakness in others, and (c) a sharp decline in performance in quantitative subjects after acceleration. No other pattern (e.g., a “highly progressive” pattern where the student shows improvement after acceleration in all areas) was observed in this preliminary sample.

Accordingly, these three patterns were chosen as the main study classification criteria because they encompass the full range of observed variance relevant to acceleration outcomes. Their selection is justified by their correspondence to different qualitative experiences following acceleration: successful concordance (highly equivalent), domain-specific inconcordance (unequal), and potentially detrimental inconcordance associated with significant academic decline (undecreasingly unequal). The “increasing” pattern—where a student performs poorly before acceleration and then improves afterward—was not included because it was not observed in the preliminary data. Furthermore, the Saudi National Acceleration Program typically only considers students with strong and consistent academic records prior to acceleration, making the low-performance pre-acceleration pattern extremely rare. Therefore, the three profiles reflect consistent experiential patterns and directly relevant to the research question.

To provide readers with a concrete understanding of the individuals under investigation, basic characteristics of each case are summarized below:

**Case 1 (M.F.)** is a female student aged 16 years at the time of data collection. She was accelerated from the 8th grade to the 10th grade, skipping the 9th grade entirely. The acceleration occurred two years prior to this study, at the age of 14. At the time of the study, she was enrolled in the 10th grade. Her profile was classified as “Even High,” characterized by uniformly high and consistent scores across all cognitive and academic domains.

**Case 2 (S.L.)** is a female student aged 17 years at the time of data collection. She was accelerated from the 9th grade to the 11th grade, skipping the 10th grade. The acceleration occurred one year prior to this study, at the age of 16. At the time of the study, she was enrolled in the 11th grade. Her profile was classified as “Uneven,” characterized by significant variability between high verbal abilities and below-average quantitative and nonverbal reasoning, with corresponding academic strengths in some subjects and marked weaknesses in others.

**Case 3 (T.M.)** is a male student aged 17 years at the time of data collection. He was accelerated from the 8th grade to the 10th grade, skipping the 9th grade. The acceleration occurred two years prior to this study, at the age of 15. At the time of the study, he was enrolled in the 10th grade. His profile was classified as “Declining Uneven,” characterized by average verbal and quantitative scores alongside a much lower-than-average nonverbal reasoning score, which was associated with a sharp decline in academic performance in mathematics and physics following acceleration.

The three cases were initially selected based on their academic performance patterns over the three semesters prior to the study. Analysis of their academic records revealed three distinct trajectories: (a) consistently high performance across all subjects (Even High), (b) high performance in some subjects alongside persistent weakness in others (Uneven), and (c) a sharp decline in performance in quantitative subjects following acceleration (Declining Uneven). Following this initial classification, the Cognitive Abilities Test (CogAT) was administered to each participant. The CogAT results subsequently confirmed these classifications, demonstrating strong convergence between the academic trajectory patterns and the cognitive profiles. Specifically, the student with the Even High academic profile exhibited uniformly high scores across all CogAT batteries; the student with the Uneven academic profile showed a pronounced discrepancy between high verbal scores and low quantitative/nonverbal scores; and the student with the Declining Uneven academic profile demonstrated a significant weakness in nonverbal reasoning that aligned with the observed decline in mathematics and physics performance. This convergence between academic and cognitive data supports the validity of the profile categorizations.

In addition to the students, the study sample also included each student’s core subject teachers (mathematics, biology, physics, chemistry, and computer science) to collect multi-source data for triangulation purposes. This allowed the researcher to assess the psychosocial aspects as well as the supportive environment. This selection reflects the sampling with the greatest variation in the key attribute of interest to the study: the degree of congruence between cognitive ability, academic performance, and the suitability of the acceleration program. This study seeks to explore how acceleration can be implemented in the Saudi context. By selecting cases that exhibit conflicting results, the analysis can provide a rich and realistic understanding of the challenges and opportunities involved. The findings may highlight both best practices and systemic weaknesses—for example, the risk of promoting students with disproportionate profiles without adequate support. However, given that this is a small-scale exploratory case study (number of cases = 3), these observations are intended to generate research questions and enrich the discussion, not to provide conclusive evidence for policy changes.

The study involved fifteen teachers, three school counselors, and six parents of gifted students. The confidentiality of all participants was maintained, and pseudonyms were used throughout the study. The Center for Gifted Students in Al-Ahsa was contacted and provided with a list of accelerated students and schools, allowing for easier communication and scheduling of assessment sessions and interviews.

### 4.3. Instrumentation

#### 4.3.1. The Cognitive Abilities Test CogAt^7^

The CogAT (Cognitive Abilities Test) Form 7, created by Lohman and Lakin (2017), seeks to determine students’ academic abilities and aptitudes in the verbal, quantitative, and nonverbal/spatial areas. The test has three cognitive subtests: verbal, quantitative, and nonverbal. The CogAT is based on the hypothesis of fluid and crystallized intelligence, which has evolved from a broad single-factor concept to a complex model.

Fluid intelligence, or fluid ability, represents natural cognitive and analytical abilities related to neurological development. It is usually unaffected by learning or cultural variables and is most visible when an individual confronts unexpected and unfamiliar settings. Crystallized intelligence, on the other hand, refers to the ability to understand relationships and solve problems using accumulated information, life experiences, and learning—whether obtained in an academic context, an educational institution, or elsewhere (A. Aboud & AlAli, 2023).

The Cognitive Abilities Test (CogAT) assesses cognitive ability across three batteries: verbal, quantitative, and nonverbal. The Verbal Battery measures verbal reasoning skills, vocabulary knowledge, and inductive and deductive reasoning through three subtests: Verbal Classification, Sentence Completion, and Verbal Analogies. The Quantitative Battery measures quantitative skills and numerical problem-solving abilities. It includes three subtests: Quantitative Relations, Number Series, and Equation Building. The Nonverbal Battery measures cognitive development, logical thinking, and problem-solving skills using geometric figures. The tests in this battery are considered culture-fair, as they do not rely on language or prior academic knowledge, thus aiming to ensure equal opportunities for all students. It includes three subtests: Figure Classification, Figure Analogies, and Figure Analysis (Lohman & Lakin, 2017).

Importantly, the cultural appropriateness and psychometric validity of the CogAT for use in the Saudi context have been established by A. Aboud and AlAli (2023), who conducted a validation study of the test with Saudi children. Their findings confirmed that the CogAT demonstrates adequate reliability and validity for assessing cognitive abilities among Saudi students, supporting its use in this study. This validation addresses concerns regarding the applicability of internationally developed assessment tools in the Saudi educational context.

#### 4.3.2. Psychosocial and Dispositional Assessment Scale (PDAS)

To evaluate the non-cognitive factors that may influence academic acceleration decisions, the researcher adapted the Psychosocial and Dispositional Assessment Scale (PDAS) from the Iowa Acceleration Scale (IAS), Third Edition (Assouline et al., 2009), with modifications to ensure cultural and contextual appropriateness for the Saudi educational setting. The IAS is a well-established, evidence-based instrument designed to guide decision making regarding whole-grade acceleration by systematically evaluating critical factors such as cognitive ability, academic performance, emotional maturity, social adjustment, and family support. Rather than creating a new scale de novo, the PDAS represents a contextual adaptation of this existing, validated instrument.

The adaptation process involved several steps to ensure relevance and appropriateness for the Saudi context. First, items from the IAS were reviewed for cultural relevance, with some items rephrased to reflect local educational practices and social norms while preserving the original construct intent. Second, the scale was translated into Arabic and reviewed by a panel of three bilingual experts in gifted education to verify accuracy and conceptual equivalence. Third, the adapted scale was piloted with a small group of teachers (n = 8) to assess clarity and applicability.

The PDAS aims to provide a systematic assessment of the emotional maturity, social adaptation, compatibility, and family support of accelerated students over a full academic year. Additionally, it seeks to identify psychosocial strengths and weaknesses that may have a significant impact on the success of a comprehensive decision-making process focused on academically accelerating gifted students and determining its suitability.

The scale consists of 17 items distributed across four core dimensions, each assessed by the student’s teacher(s) on a four-point Likert scale of frequency (1 = rarely, 2 = sometimes, 3 = often, 4 = always). The four dimensions are: Emotional Growth (4 items): Measures emotional regulation, resilience, and responsiveness to feedback and challenges; Peer Relationships (5 items): Assesses social skills, quality of friendships, and ease of integration with peers of the same age and older; Student Attitude towards Full Grade Acceleration (4 items): Measures the student’s level of self-motivation, enthusiasm, and concerns about acceleration; and Parental Attitude and Support (4 items): Measures the degree and quality of parental involvement, advocacy, and support for the acceleration process.

The PDAS was administered to the core subject teachers of each case study participant. The multiple assessment approach provided a composite and crosscutting perspective on the psychosocial readiness of accelerated students, complementing objective cognitive and achievement data.

Because the PDAS is a culturally adapted version of the Iowa Acceleration Scale (IAS; Assouline et al., 2009), the validity evidence was compiled through the integrative methodology of theoretical (logical) validity and content validity:

*Theoretical (Logical) Validity*

The PDAS was not developed from scratch but is a contextual adaptation of the Iowa Acceleration Scale, a well-established and evidence-based tool used for over two decades in acceleration decision making (Assouline et al., 2009; 2015). The original scale has been validated through extensive field testing and multiple replication studies, demonstrating its usefulness in predicting successful acceleration outcomes (Steenbergen-Hu et al., 2016). By retaining the core structures of the original scale while modifying only the linguistic and cultural formulations, the PDAS inherits significant theoretical validity from the original scale. This alignment ensures that the PDAS measures the same underlying constructs—emotional maturity, social adjustment, student motivation, and family support—that have been consistently identified in the literature as critical to acceleration success (Colangelo et al., 2004).

*Content Validity*

Content validity refers to the extent to which the items of a scale adequately represent the intended theoretical construct (Haynes et al., 1995). To establish content validity, the 17 items of the scale were independently reviewed by five experts in gifted education and educational psychology (three university faculty members and two experienced practitioners in the field of academic acceleration). Each expert rated the relevance of each item to its assigned dimension using a 4-point scale (1 = not relevant, 4 = highly relevant). The Item-Content Validity Index (I-CVI) was calculated for each item, and the Scale-Level Content Validity Index (S-CVI) was computed as the proportion of items rated as relevant (ratings of 3 or 4) by all experts (Polit & Beck, 2006).

The results indicated that all items achieved an I-CVI of 0.80 or higher, with a mean S-CVI of 0.94, exceeding the recommended threshold of 0.90 (Polit & Beck, 2006). This provided strong evidence that the PDAS items are relevant and representative of the four intended psychological dimensions.

Reliability analysis yielded satisfactory internal consistency for all dimensions: Emotional Growth (Cronbach’s α = 0.792), Peer Relationships (α = 0.893), Student Attitude (α = 0.881), and Parental Attitude and Support (α = 0.871), all exceeding the acceptable threshold of 0.70 (Assouline et al., 2009). While these values indicate good internal consistency, the adapted nature of the instrument is acknowledged as a limitation, and future research should conduct more comprehensive validation procedures, including confirmatory factor analysis.

#### 4.3.3. Interviews

Semi-structured interviews formed the integrative explanatory pillar within this multi-curriculum design. While cognitive (CogAT), academic (grade records), and psychosocial (PDAS) data provided standardized and quantifiable measurements of the outcomes that occurred, the interviews provided an explanatory function by revealing the cause and manner of these outcomes from the perspective of students, teachers, and parents.

Interviews were chosen as an integrative tool because they are the only instrument that covers all three perspectives of each case, thus enabling triangulation of interpretations. They allow for the exploration of related and dissimilar themes that quantitative measures alone cannot capture. Furthermore, they bring contextual meaning to statistical patterns, enabling, for example, a student with an uneven cognitive level to explain why they are experiencing emotional distress, rather than simply documenting its presence. Therefore, interviews are described as “essential” in their explanatory function within the integrative analysis, not in the sense of replacing quantitative data.

#### 4.3.4. Limitations of Instruments and Mediating Efforts

Each instrument used in this study has potential limitations, and deliberate steps were taken to mitigate them.

CogAT Test: Although the CogAT is a well-standardized measure of cognitive abilities, it was primarily developed in the United States and may carry cultural biases. To mitigate this, the study used the version validated for Saudi children by A. Aboud and AlAli (2023), which confirms its cultural appropriateness and psychometric reliability. Furthermore, CogAT scores were never interpreted in isolation; rather, they were triangulated with academic records and qualitative interviews to avoid over-reliance on a single cognitive measure.

The PDAS (adapted from the Iowa Acceleration Scale): As a culturally adapted tool, the PDAS may not fully capture all culturally specific aspects of psychosocial readiness in Saudi Arabia. To mitigate this, the adaptation process included review by bilingual experts, initial pilot testing with Saudi teachers, and calculation of content validity and internal consistency indices. Furthermore, the PDASs was supplemented with in-depth qualitative interviews with students, parents, and teachers, which provided rich contextual information that could not be captured by Likert scales alone.

Semi-structured interviews, which rely on self-reporting and retrospective narratives, are susceptible to recall bias, social desire bias, and interpreter subjectivity. To mitigate these limitations, the study employed multi-perspective triangulation (students, teachers, parents, and counselors) for cross-validation. Objective analysis was conducted with two independent coders, achieving a high degree of inter-coder agreement (Cohen’s kappa = 0.88) to minimize interpretive bias.

## 5. Data Analysis

### 5.1. Predictive Linear Model for Academic Performance Analysis

To evaluate acceleration outcomes in a more informative manner than raw pre-post grade differences, this study employed a descriptive predictive heuristic. The term “predictive” here is used descriptively (not inferentially): the model generates an individualized benchmark that accounts for (a) each student’s pre-acceleration performance stability and (b) the typical grade-level difficulty increase observed among non-accelerated peers. This benchmark allows the researcher to describe whether a student’s post-acceleration performance fell above, at, or below a context-adjusted expectation.

### 5.2. Model Specification and Calculation

The heuristic was operationalized as: E = (A × ω) + G

Where E = Expected performance (benchmark percentage grade), A = Student’s three-semester average prior to acceleration, and ω = Individually calibrated coefficient of stability (range 0.85–0.97), derived from the standard deviation of pre-acceleration grades. G = Grade-level adjustment factor (range +2 to +5 percentage points), derived from three years of school records for non-accelerated students

The coefficient of stability (ω) was calibrated as follows: students with highly consistent performance (SD < 3%) received ω = 0.95–0.97; moderate variability (SD 3–7%) received ω = 0.88–0.94; high variability (SD > 7%) received ω = 0.85–0.87. These thresholds are descriptive conventions, not statistically derived cutoffs.

The grade-level adjustment factor (G) was calculated from the observed mean grade decline between consecutive grade levels among 120 non-accelerated students in the same schools. For single-grade skips, G ranged from +2 to +3 points; for two-grade skips, +4 to +5 points.

### 5.3. Descriptive, Not Inferential

The performance gaps calculated from this model are descriptive only. They are not subjected to statistical significance tests, as the case study methodology relies on analytical generalization rather than statistical inference (Yin, 2018), and the sample size (N = 3) does not permit meaningful inferential analysis. These gaps serve as exploratory heuristics—tools to organize observations—and are interpreted strictly in conjunction with cognitive (CogAT), psychosocial (PDAS), and qualitative interview data.

An illustrative example: A student with a pre-acceleration three-semester average of 88% in mathematics and moderate performance variability (SD = 4.5%) receives ω = 0.92. Skipping one grade (G = +3) yields: E = (88 × 0.92) + 3 = 84%. If the student achieves 77% post-acceleration, the descriptive gap is −7 points.

### 5.4. Model Validation

The model was calibrated using data from 120 non-accelerated students (grades 8–12). Cross-validation on a holdout sample of 40 non-accelerated students produced root mean square errors of 3.2–4.8 percentage points across subjects. This indicates that, for non-accelerated students in this context, the model has acceptable descriptive accuracy. However, this validation does not establish causal validity for accelerated students.

### 5.5. Triangulation and the Problem of Confounding Variables

As an important limitation, the model does not—and cannot—control for potential confounding variables such as life events (e.g., family disruption, illness), developmental changes associated with puberty, peer group dynamics, changes in educators or teaching quality, or fluctuations in home support systems. Any observed performance gap could, in principle, be attributed to these factors rather than to acceleration itself.

In the three cases presented, no informant reported any significant life event, educator change, or peer conflict that could plausibly account for the observed patterns. This absence of alternative explanations strengthens the descriptive interpretation, but it does not permit causal claims. Causality cannot be inferred from this case study design.

## 6. Results

### 6.1. How Can an Integrated, Case-Study-Based Assessment Model Guide Decision Making to Accelerate Academically Gifted Students?

The three cases were initially identified based on their academic performance patterns over the three semesters prior to the study. The Cognitive Abilities Test (CogAT) was subsequently administered to validate and deepen understanding of these classifications. The following sections present the following for each case: (a) the initial academic profile that informed selection, (b) the CogAT results that confirmed the cognitive basis of that profile, and (c) the integrated analysis of cognitive, academic, and psychosocial data that informed the acceleration decision.

#### 6.1.1. Case Study 1 (M.F.)

Prior to the study, analysis of M.F.’s academic records over the three preceding semesters revealed a pattern of consistently high performance across all subjects, with grades ranging from 94% to 100% in mathematics, biology, physics, chemistry, and computer science. This pattern was classified as “Even High.”

**Cognitive Ability Assessment (CogAT)**

Following selection, the CogAT was administered to validate this classification. Table 1 displays M.F.’s CogAT profile.

As shown in Table 1, M.F. demonstrates an “Even High” cognitive profile, characterized by uniformly high scores across all three batteries with minimal variability (standard deviation of SAS scores = 7.6 points). Her composite SAS of 148 places her at the 99th percentile nationally. According to the interpretive framework of the CogAT (Lohman & Lakin, 2017), such a profile suggests well-integrated intellectual resources, balanced cognitive strengths, and the capacity for rapid learning when appropriately challenged. **No claims about specific learning behaviors or social preferences are derived from Table 1 alone**; those are reported below from qualitative and psychosocial data.

Academic Achievement Assessment

A predictive linear model (Section 5.1) was used to generate individualized expected performance benchmarks. Table 2 presents the analysis.

**Figure 1:**
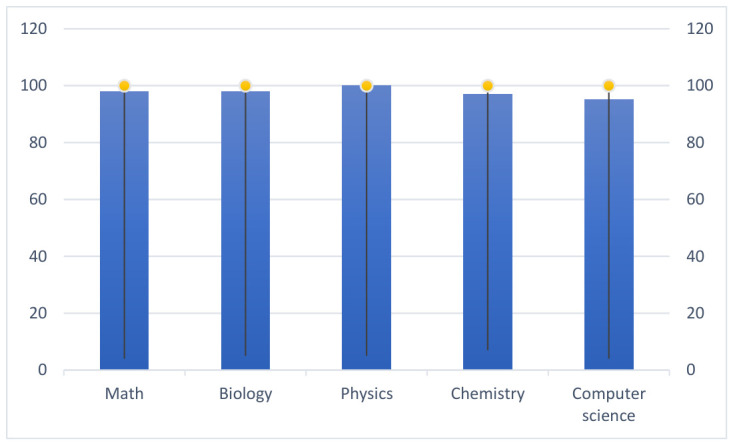
Even profile of student M.F.

As shown in Table 2, M.F. consistently exceeded model expectations across all subjects, with an average positive performance gap of +5 points. Her sustained high achievement (grades 95–100%) validates the “Even High” pattern identified in the cognitive assessment. The largest positive gap (+7 in Chemistry) indicates that she performed substantially above the expected benchmark in a subject often considered challenging. Figure 1 shows M.F.’s even profile.

Teacher interview data confirmed these academic findings. Her mathematics teacher stated: “M.F. has exceptional cognitive development, mathematical prowess, and a high level of motivation to tackle challenging tasks.” Another teacher added: “M.F. has an outstanding memory and a remarkable ability to transfer and apply knowledge to solve new problems.”

Student interview: When asked how skipping a grade affected her studies, M.F. responded: “I became more motivated and challenged. Before I was accelerated, I felt bored because I would rush through assignments and have to wait for my classmates. Now, I feel the assignments challenge me, and I don’t feel like my time is being wasted.”

Parent interviews: M.F.’s mother said: “We are very proud of M.F. When the school suggested acceleration, we felt some apprehension, like any parents, but we saw her passion and enthusiasm, and it was the right decision.” Her father added: “I was in favor of acceleration because I saw that she had not progressed in her old class. Boredom was evident in her behavior. Currently, she has integrated with peers who challenge her intellectually.”

Psychosocial and Dispositional Assessment (PDAS)

Teachers completed the PDAS to assess M.F.’s emotional maturity, social adjustment, and attitude toward acceleration. Table 3 summarizes the ratings.

As shown in Table 3, teachers rated M.F. highly on all dimensions. She demonstrates maturity in emotional regulation, shows no sensitivity or aggression toward feedback, and reflects on criticism to adjust her behavior. Regarding peer relationships, teachers noted that she has well-developed social skills appropriate for her age and interacts effectively with both same-age and older peers, though she does prefer the company of older students—a preference consistent with her cognitive maturity. Her attitude toward acceleration is very favorable, with strong intrinsic motivation. Parental support was rated as extremely supportive and committed, with no evidence of excessive pressure or neglect.

Student interview (emotional and social aspects): M.F. stated: *“Before acceleration, I constantly felt frustrated and bored, and I often felt angry or sad for no clear reason. Now these feelings have almost disappeared because my mental energy is occupied with something satisfying*. *My parents support me. They ask about my studies and celebrate my achievements, but they don’t comment on every single grade.”* Regarding peer relationships, she said: *“I finally feel like I’m in the proper place; my bond with my new classmates has deepened, making me feel at ease and understood.”*

Integration and Acceleration Decision—Student M.F.

The case study data for student M.F. clearly converges in three areas. Cognitively, the student demonstrates a consistently high level on the CogAT, with minimal variation across all tests, and a total SAS score of 148 (99th percentile).

Academically, her performance after the acceleration program consistently exceeded typical expectations, with an average positive difference of 5 points in all subjects, and maintained grades between 95% and 100%. Psychosocially, she received high ratings in all dimensions of the Personal Functioning Assessment Scale (PDAS), demonstrating her emotional maturity, positive peer relationships, strong motivation, and family support. In interviews, she reported increased satisfaction and a greater sense of belonging in the acceleration program she attended.

Based on the convergence of these three pieces of evidence, the researcher concludes that student (M.F.) is an ideal candidate for grade-wide acceleration. Her balanced cognitive status indicates that she possesses the intellectual resources necessary to meet the demands of the accelerated curriculum. Her academic performance demonstrates that the intervention has been academically beneficial. Her psychosocial readiness—in terms of emotional regulation, positive peer relationships, strong self-motivation, and family support—indicates that she possesses the non-cognitive resources necessary to cope with the social and emotional dimensions of academic acceleration. However, ongoing monitoring remains essential to ensure continued adjustment over time.

#### 6.1.2. Case Study 2 (S.L.)

Prior to the study, analysis of S.L.’s academic records over the three preceding semesters revealed a pattern of high performance in some subjects (Computer Science, Biology) alongside persistent weakness in others (Mathematics, Physics, Chemistry). This pattern was classified as “Uneven.”

Cognitive Ability Assessment (CogAT)

Following selection, the CogAT was administered to validate this classification. Table 4 displays S.L.’s CogAT profile.

As shown in Table 4, S.L. exhibits a highly **uneven** cognitive profile. Her verbal reasoning ability is relatively high (SAS = 124, PR = 93), but her quantitative reasoning and nonverbal reasoning scores are significantly lower (SAS = 75, PR = 6 for both). The discrepancy between her verbal and quantitative scores is 49 SAS points, and the standard deviation across the three batteries is 28.2—substantially exceeding the 1.5 threshold that indicates significant profile variability. Her composite score (SAS = 89, PR = 25) masks these critical discrepancies. No claims about learning behaviors or academic struggles are derived from Table 4 alone; those are reported below from academic, interview, and psychosocial data.

Academic Achievement Assessment

Table 5 presents S.L.’s academic performance, comparing current post-acceleration grades to model-predicted expectations.

**Figure 2:**
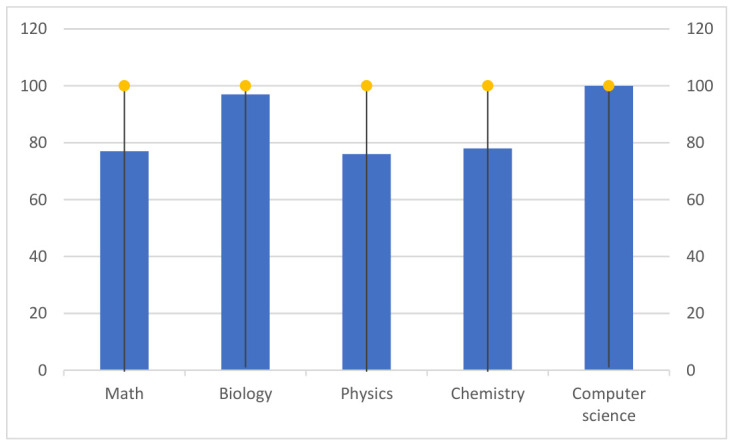
Uneven profile of student S.L.

As shown in Table 5, S.L.’s academic performance mirrors her uneven cognitive profile. She excels in Computer Science (100%, top 1%) and Biology (97%, top 4%), but her performance in Mathematics (77%), Physics (76%), and Chemistry (78%) is substantially lower—below her own pre-acceleration averages and below model expectations (negative gaps ranging from −1 to −4 points). Figure 2 shows the uneven profile of student S.L.

Teacher interview data confirmed this pattern. One teacher stated: *“The student excels at computer science and biology, but struggles with mathematics and physics.”* Another teacher added: *“Acceleration widened the gap for the student because the curriculum assumes she is capable of everything at the same level, and this did not apply to the student.”*

Student interview: When asked how skipping a grade affected her studies, S.L. responded: *“I used to feel confident about myself academically, but after the acceleration, especially in mathematics, I started to doubt my abilities. The low grades made me afraid of these subjects instead of loving them.”*

Parent interview: S.L.’s mother said: *“My daughter is a genius in computers and loves biology, but her performance in the other subjects is not at the same level. We believe that with a little help, her performance will improve.”*

Psychosocial and Dispositional Assessment (PDAS)

Teachers completed the PDAS to assess S.L.’s emotional maturity, social adjustment, and attitude toward acceleration. Table 6 summarizes the ratings.

As shown in Table 6, teachers reported that S.L. sometimes exhibits emotional disturbance and is often highly sensitive to criticism. Although she is capable of considering feedback, her sensitivity and occasional defensive reactions suggest difficulties in emotional regulation, potentially exacerbated by academic frustration.

Teacher comment (mathematics teacher): *“Despite S.L.’s excellence in some subjects, her feeling that she is not the best in her new class—compared to how she was before acceleration—sometimes leaves her frustrated and downhearted.”*

Student interview (emotional and social): S.L. stated: *“I feel that every comment from my teacher is a personal attack and a sign that I am incompetent. That’s what makes me get angry quickly.”* Regarding peer relationships, she said: *“I feel like a stranger in both places. My old classmates see me as ‘the advanced one’ who left them, and my new classmates are older and have different interests. Sometimes I feel like I don’t belong to any group, and it’s a very lonely feeling.”*

Student attitude toward acceleration: When asked about her view on acceleration, S.L. said: *“Sometimes I think the acceleration decision was terrible. I feel constant pressure because the curriculum advances so quickly, and if I miss even a little point, I feel like I’m falling behind my classmates.”*

Parental support: Teachers noted that parental involvement sometimes tends toward excessive interference, placing pressure on the student. One teacher commented: *“There is general parental support, but it is mixed with excessive pressure, which contributes to the student’s anxiety and inconsistent performance.”* S.L. shared: *“I feel anxious and afraid of how my parents will react to my results in some subjects*. *I study out of fear of failure or disappointing expectations.”* Her mother acknowledged: *“Sometimes I push her to study more because I am afraid that she will neglect the difficult subjects and fall further behind. I know that I am putting pressure on her, but my fear for her drives me to do so.”* The mother also expressed worry: *“I am very worried about her. I see her exhausted, frustrated, and struggling to keep up with her peers. She is no longer that cheerful child. I want her to withdraw from the acceleration because she is paying a heavy psychological price. But her father sees it as a failure. We argue, so I no longer know what the right decision is.”* The father stated: *“We spend a lot of money on private lessons. The problem is not speeding up; the problem is her lack of focus!”*

Integration and Acceleration Decision—Student S.L.

The data from Student S.L.’s case study reveal consistent patterns across cognitive, academic, psychological, and social domains. Cognitively, the student exhibits significant variability in her CogAT scores: high verbal ability (SAS = 124, 93rd percentile) coupled with remarkably low scores in quantitative and nonverbal reasoning (SAS = 75, 6th percentile for both), with a 49-point difference between her highest and lowest SAS scores in these two domains.

Academically, her performance reflects this cognitive disparity; she excels in subjects requiring intensive language skills, such as computer science and biology, but underperforms in quantitative subjects like mathematics, physics, and chemistry. Psychologically and socially, while she demonstrates high motivation in her areas of strength, she also suffers from emotional distress, hypersensitivity to criticism, social isolation, and conflicting parental pressures.

Based on the convergence of these three pieces of evidence, the researcher concludes that full academic acceleration is not appropriate for S.L.’s needs. Her academic profile suggests that the most suitable intervention is acceleration in specific subjects—limited to computer science and biology, where she excels—along with targeted academic intervention in mathematics, physics, and chemistry to address her weaknesses. Psychosocial support focused on emotional regulation and building self-confidence is also strongly recommended. This interpretation is offered with the understanding that ongoing monitoring and periodic adjustments are essential to ensure her well-being and academic progress.

#### 6.1.3. Case Study 3 (T.M.)

Prior to the study, T.M.’s academic records over the three preceding semesters showed a pattern of high performance across most subjects, with averages of 84% in mathematics and 90% in physics. However, following acceleration, his performance declined sharply to 47% in mathematics and 45% in physics, while remaining strong in biology, chemistry, and computer science. This pattern was classified as “Declining Uneven.”

Cognitive Ability Assessment (CogAT)

Following selection, the CogAT was administered to validate this classification. Table 7 displays T.M.’s CogAT profile.

As shown in Table 7, T.M. presents an uneven cognitive profile. His verbal (SAS = 102, PR = 55) and quantitative (SAS = 103, PR = 57) scores fall within the average range. However, his nonverbal reasoning score (SAS = 84, PR = 16) is significantly lower, representing a relative weakness in fluid reasoning—the ability to solve novel problems, recognize patterns, and reason spatially independent of learned knowledge. The discrepancy between his quantitative and nonverbal scores is 19 SAS points, with a standard deviation of 10.6 across the three batteries. His composite score (PR = 40) masks this domain-specific weakness. No claims about academic decline or learning behaviors are derived from Table 7 alone; those are reported below from academic, interview, and psychosocial data.

Academic Achievement Assessment

Table 8 presents T.M.’s academic performance before and after acceleration.

**Figure 3:**
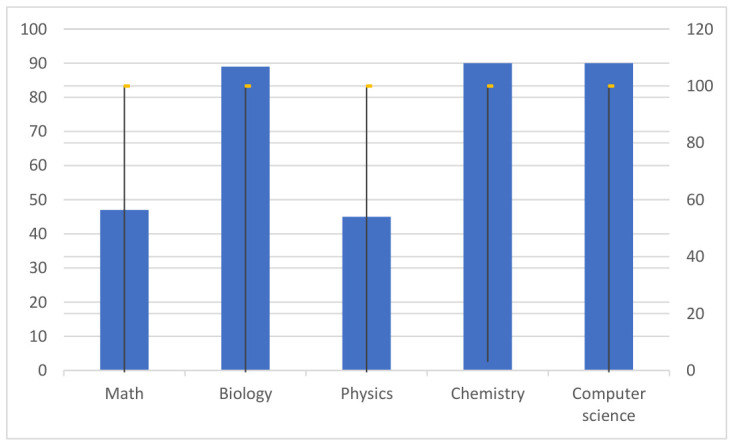
Uneven profile of student T.M.

As shown in Table 8, T.M.’s profile is critically uneven. He maintains strong performance in biology (89%), chemistry (90%), and computer science (90%)—subjects that rely more heavily on verbal and quantitative knowledge. However, he shows a precipitous drop in mathematics (from 84% to 47%, a −37-point gap) and physics (from 90% to 45%, a −45-point gap). These are the subjects that demand fluid reasoning and spatial-analytical thinking—domains in which his CogAT nonverbal score indicates relative weakness. This uneven pattern is also illustrated in Figure 3.

Teacher interview data confirmed this pattern. The mathematics teacher stated: *“Student T.M. has clear challenges and significant difficulties in connecting abstract concepts. He can memorize steps, but he finds it very difficult to apply them to new problems.”* The physics teacher added: *“I observe a stark gap between T.M.’s ability to grasp theory and his inability to apply it in solving physics problems.”*

Student interview: T.M. acknowledged: *“I have serious difficulties in mathematics. I need help and support to understand some concepts and math problems.”* When asked whether subjects became easier or harder after acceleration, he said: *“After my acceleration, I was forced to seek help and take private tutoring in mathematics just to keep up with my classmates.”* He also revealed: *“I have an anxiety about physics that causes me to ‘freeze’ intellectually when faced with any practical application.”*

All of T.M.’s teachers expressed concern about his continuation in the acceleration program. One stated: *“I believe the decision to accelerate should be reviewed; he is always stressed and fatigued from trying to keep up with his peers.”*

Psychosocial and Dispositional Assessment (PDAS)

Teachers completed the PDAS to assess T.M.’s emotional maturity, social adjustment, and attitude toward acceleration. Table 9 summarizes the ratings.

As shown in Table 9, T.M. frequently displays emotional instability and is often highly sensitive to criticism. He sometimes reacts with aggression or defensiveness. His social skills are rated as less developed than those of his peers, with few friendships and difficulty integrating into his accelerated study group.

School counselor report: *“What concerns me more than the grades is the change in T.M.’s psychological and social state. I’ve noticed obvious withdrawal, irritation, and deep frustration. Acceleration has not provided him with a stimulating challenge; rather, it has presented him with repeated experiences of failure in specific areas, threatening his self-esteem and overall motivation to learn. The emphasis should immediately change from ‘catching up with the curriculum’ to regaining his confidence as a learner.”*

Student interview (emotional and social): T.M. stated: *“I feel psychological pressure and am unable to keep up with my classmates.”* Regarding his attitude toward acceleration, he said: *“I don’t regret accelerating; it was a beneficial experience that showed my inadequacies, which I am working hard to overcome.”* However, his teacher disagreed, as noted above.

Parental support and conflict: T.M. expressed fear of failure and inability to meet parental expectations: *“‘You are lazy; you need to work harder.’ They refused to recognize that acceleration could be part of the problem. They made me believe that the failure was entirely my fault, which compounded my feelings of powerlessness and guilt.”* He added: *“My mother is concerned and wants me to reverse the acceleration for my psychological well-being. I feel like I’m a battleground for them, and no one asks what I want. There is no space for error or experimenting. I must strictly adhere to their plan, or else I am ‘uncooperative’ or ‘a quitter.’”*

Integration and Acceleration Decision—Student T.M.

The data from student T.M.’s case study are consistent across cognitive, academic, psychological, and social domains. Cognitively, he shows inconsistent performance on the CogAT: his verbal and quantitative scores are within the average range, but he demonstrates significant weakness in nonverbal reasoning (16th percentile), indicating difficulties with fluid reasoning—that is, the ability to solve novel problems, recognize patterns, and reason spatially independently of acquired knowledge.

Academically, his performance after the accelerated program was highly inconsistent: while he maintained strong results in biology, chemistry, and computer science, he experienced a sharp decline in mathematics (a 37-percentage point drop) and physics (a 45-percentage point drop)—precisely the subjects that require flexible reasoning. Psychologically and socially, he exhibits emotional instability, hypersensitivity to criticism, social withdrawal, and conflicting views with his parents; his school counselor has expressed serious concern about his mental health.

Based on the convergence of these three pieces of evidence, the researcher believes that accelerating student (T.M.) academically necessitates reconsidering the granting of full academic excellence. His marked weakness in logical reasoning is perfectly aligned with his sharp decline in mathematics and physics, two subjects that heavily rely on this type of thinking. Furthermore, his psychological distress coincides with his academic difficulties, and the convergence of cognitive, academic, and psychosocial factors indicates a clear incompatibility with the full academic acceleration program.

Accordingly, the researcher recommends considering reintegrating the student into an age-appropriate class, while offering enrichment programs in his areas of strength (biology, chemistry, and computer science) as a viable and beneficial alternative. Should the accelerated learning continue despite this evidence, a comprehensive support plan becomes essential. This plan should include targeted academic intervention in mathematics and physics, social and psychological support to restore his self-confidence, and guidance for parents to address any conflicting expectations. This interpretation is offered with the understanding that the case study design does not allow for causal inferences, and that ongoing follow-up is necessary regardless of the enrollment decision.

## 7. Discussion

The three case studies analyzed in this research were chosen to illustrate the role of alignment—or misalignment—between a student’s cognitive profile, academic performance, and psychosocial support system in the context of academic acceleration decisions. Because this is a small-scale qualitative case study (number of cases = 3), statistical generalization is not possible (Yin, 2018). The observed patterns are suggestive rather than definitive and are presented to generate research questions and provisional insights for future investigation rather than to confirm existing theories (Assouline et al., 2015; Steenbergen-Hu et al., 2016).

### 7.1. Comparative Patterns Between the Cases

When the three cases were systematically compared, distinct patterns emerged. Student M.F. (high even profile) demonstrated consistently strong cognitive abilities across all CogAT domains (SAS range 131–146, percentile 97–99), with minimal variance. Her academic performance consistently exceeded typical expectations across all subjects (positive gap average +5 points), and she maintained high achievement (95–100%). Psychosocially, she scored highly on the PDAS for emotional maturity, peer relationships, and motivation, with strong and consistent parental support. In interviews, she reported increased motivation and satisfaction.

Student S.L. (uneven profile) exhibited a stark contrast: high verbal ability (SAS = 124, percentile = 93) coupled with low quantitative and non-verbal scores (SAS = 75, percentile = 6 for both). Her academic performance reflected this pattern: excelling in verbally intensive subjects (Computer Science, Biology) alongside difficulties in quantitative subjects (Mathematics, Physics, Chemistry), with performance gaps ranging from −1 to −4 points in those areas. Psychosocially, she reported high motivation in her areas of strength but experienced emotional distress, hypersensitivity to criticism, social isolation, and conflicting parental pressure.

Student T.M. (Declining Uneven) submitted average verbal and quantitative scores (SAS = 102–103, percentile 55–57) but with significant weakness in nonverbal reasoning (SAS = 84, percentile 16). His academic record prior to acceleration was seemingly strong (84% in mathematics, 90% in physics), but after acceleration he showed a sharp decline in mathematics (−37 points) and physics (−45 points), while maintaining strong performance in biology, chemistry, and computer science. Psychologically and socially, he exhibited emotional instability, social withdrawal, and conflicting parental views, with the school counselor expressing deep concern.

Interpretive reasoning: These patterns suggest that students with different cognitive profiles may have different acceleration experiences. However, because there is no control group and no baseline measurements of psychosocial performance before acceleration, it is not possible to determine whether the observed differences are caused by the acceleration or pre-existing. Alternative explanations—including prior differences in temperament, family dynamics, classroom environment, or teaching quality—cannot be ruled out. Therefore, the observed patterns should be interpreted as exploratory and generative of research questions for future investigation.

### 7.2. Composite Scores as Misleading Indicators

The finding of the study showed that in each of the three cases, a composite measure—whether a CogAT composite score or an overall academic average—provided a different picture than the subtest or domain-specific scores. For Student M.F., the composite score (SAS = 148) was consistent with her subtest scores. For Student S.L., an average composite CogAT score (SAS = 89) masked a 49-point discrepancy between her superior verbal abilities (SAS = 124) and significant weaknesses in quantitative and nonverbal reasoning (SAS = 75 for both). For Student T.M., the composite CogAT score (PR = 40) concealed a pronounced deficit in fluid reasoning, as measured by the Nonverbal battery (PR = 16), and this deficit was followed by a steep academic decline in mathematics and physics after acceleration.

These observations are consistent with the argument previously made by scholars such as Lohman and Lakin (2017) that composite scores can be misleading by averaging out important individual differences. However, because only three cases are presented, it is not possible to determine how frequently such masking occurs or under what conditions composite scores are most likely to be misleading, as noted by Lohman and Lakin (2017). The inference that “a decision based on a composite score could lead to an inappropriate placement” is a logical extrapolation from these cases, not a statistical finding. Therefore, readers are advised to view this as a cautionary illustration rather than as definitive evidence.

### 7.3. Data Convergence Across Domains

For each student, data from cognitive assessments, academic records, and psychosocial assessments were examined together.

M.F.: Cognitive data (uniformly strong), academic data (sustained high performance exceeding expectations), and psychosocial data (high PDAS scores, positive interviews) all converged toward a successful agreement.

S.L.: Cognitive data (verbal strength, quantitative/nonverbal weakness), academic data (excellence in verbally intensive subjects, difficulties in quantitative subjects), and psychosocial data (high motivation in strength areas, emotional distress in weakness areas) showed a consistent pattern of domain variance.

T.M.: Cognitive data (nonverbal weakness), academic data (sharp decline in mathematics and physics after acceleration), and psychosocial data (emotional instability, social withdrawal, conflicting family) converged on difficulties following acceleration.

The convergence of data across these three domains supports the idea that a multifaceted assessment approach provides a more complete picture than any single measure alone. For M.F., the convergence of positive indicators is consistent with the likelihood that full acceleration was appropriate (Steenbergen-Hu & Moon, 2011; Y. Aboud, 2021). For S.L. and T.M., the convergence of difficulties is consistent with the likelihood that acceleration was not appropriate for their profiles. However, alternative explanations cannot be ruled out, and these observations should be considered illustrative rather than definitive.

### 7.4. The Role of Psychosocial Support

Parental support, as measured by the “Parental Attitude and Support” dimension of the PDAS, varied significantly among the three cases and was closely correlated with student outcome patterns.

In the case of student M.F., parental support was rated high, with parents being supportive and committed without exerting undue pressure. This was correlated with clearly positive academic and emotional outcomes. In the case of student S.L., parental support was moderate; parents were supportive but exhibited clear anxiety and some pressure. This coincided with mixed results and emotional distress for the student. In the case of student T.M., parental support was low, with conflicting viewpoints between the parents and psychological pressure. This was correlated with negative academic and emotional outcomes.

Qualitative interviews provided deeper context for these patterns. Student T.M. realized that his parents’ differing perspectives exacerbated his feelings of being “trapped” and “failing to meet expectations.” S.L.’s mother admitted to pushing her daughter to study harder out of fear, while T.M.’s mother wanted to reverse his decision to accelerate, a move his father viewed as a “failure.”

This striking correlation between high levels of parental support and positive student outcomes (as in the case of M.F.), and between low support and negative outcomes (as in the case of T.M.), is consistent with the broader literature emphasizing the importance of family support for the success of academic acceleration (Colangelo et al., 2004). However, interpretation should be approached with caution, as this study did not measure parental support prior to acceleration, making it impossible to determine the direction of any causal relationship. Low support may have caused the negative outcomes, or the negative outcomes may have led to less parental support, or a third variable (such as prior family conflict) may have influenced both.

The tentative inference that can be made here is that parental support and assistance in navigating the acceleration process may be just as important as supporting the student themselves—but this remains a proposition that needs to be tested in larger, controlled studies with control groups and longitudinal designs (Cross et al., 2018; Bernstein et al., 2021). Until such evidence is available, these observations should be viewed as illustrative and as generating research questions, not as conclusive or policy making in themselves.

### 7.5. Systemic Effects

Two out of three cases (S.L. and T.M.) showed significant cognitive variances (defined as a difference of 15 or more points in SAS scores between cognitive domains) and experienced academic and/or psychosocial difficulties after acceleration. The third case (M.F.) did not show such variances and did not experience any observed difficulties.

These observations suggest that current identification and placement procedures—which may rely heavily on composite academic averages and test scores—could be improved by incorporating a more comprehensive assessment of individual cognitive profiles and psychosocial readiness. However, because this inference is based on only three cases, it is not possible to establish a baseline rate of successful versus unsuccessful accelerations among students with such diverse profiles. Many students with profiles similar to S.L. or T.M. may be successful in acceleration; conversely, some students with profiles similar to M.F. may experience difficulties.

Current data cannot answer these questions (Yin, 2018). Therefore, the systemic effects presented here are tentative and speculative, intended to stimulate discussion and future research rather than guide policy changes based solely on this study. This observation aligns with calls in the literature for a more flexible and individualized approach to acceleration (Bernstein et al., 2021; Y. Aboud, 2025).

### 7.6. Summary of Interpretive Caution

No causal claims can be made from this study. Words such as “effect,” “impact,” “consequence,” or “cause” were deliberately avoided when referring to the relationship between acceleration and outcomes. Instead, the study presents observations, patterns, correlations, and participant experiences. Where interpretive inferences are made, they are explicitly identified as provisional, context-specific, and requiring replication in larger and more diverse samples.

Specifically: Positive results for M.F. should not be interpreted as evidence that acceleration is universally beneficial; nor should difficulties with S.L. and T.M. be interpreted as evidence that acceleration is universally detrimental. Other students with profiles similar to S.L. or T.M. may succeed with acceleration; other students with profiles similar to M.F. may experience difficulties. Current data cannot predict individual outcomes.

Comparative case analysis reveals an association between strong alignment across cognitive, academic, and psychosocial dimensions and successful acceleration (as observed in the M.F. case), and an association between inconsistency in one or more dimensions and adverse outcomes (as observed in the S.L. and T.M. cases). These associations are consistent with the broader body of evidence but do not independently confirm it (Steenbergen-Hu et al., 2016; Assouline et al., 2015). The findings lead us to view the explanations presented here as generative of research questions rather than as hypothesis-confirming.

## 8. Conclusions

The accelerated decision-making process, which was the primary focus of the current study, oscillated between moving forward, progressing with caution, and reconsidering based on the three case studies examined. The transition from a single model based on accelerated students’ academic performance to an integrated evaluation process centered on the student’s comprehensive academic, cognitive, and psychosocial profile—which formed the core of the study—provided a solid foundation for making the safest and most appropriate decision about whether to continue acceleration or not.

Creating flexible acceleration paths has now become a priority in Saudi Arabia and other contexts to ensure that gifted students receive effective and adaptable support, protecting their well-being while also allowing them to attain their full potential.

## 9. Study Limitations and Future Directions

The three case studies in the current study provided important insights into the decision-making process for academic acceleration of brilliant adolescents in Saudi Arabia. However, there are numerous constraints to consider. The first is the study’s small sample size: while it was based on three carefully selected cases from the Al-Ahsa region, chosen to reflect diverse cognitive and academic profiles, the findings may still be limited in their applicability to all gifted students in Saudi Arabia who are eligible for acceleration. Furthermore, the study was limited to examining accelerated students’ experiences over a specific time period; as a result, the long-term effects of acceleration choices, such as academic performance trajectory, career development, and the psychological impact of acceleration on their growth, remain unknown. Therefore, there is a need for a longitudinal study to assess the enduring impact of acceleration. Additionally, this study focused exclusively on STEM subject teachers (mathematics, biology, physics, chemistry, and computer science), reflecting the weighting of the Saudi acceleration program’s qualifying examination. Language-based subjects and the arts were not included, limiting our understanding of accelerated students’ performance and adjustment in these domains. Future research should incorporate perspectives from a broader range of subject areas to provide a more comprehensive assessment of acceleration outcomes.

Lastly, although parents’ and teachers’ perspectives on the acceleration experience are significant, they could not accurately represent the experiences of pupils who have been accelerated. Thus, more comprehensive metrics like psychological well-being, quality of life, and other standardized psychosocial evaluations may be included in future research.

## Figures and Tables

**Table 1:** CogAT profile for student M.F.

Battery	Raw Score	Standard Age Score (SAS) ^1^	Percentile Rank (PR) ^2^	Stanine (S) ^3^
Verbal	61/65	131	97	9
Quantitative	57/60	140	99	9
Nonverbal	64/65	146	99	9
Composite	182/190	148	99	9

^1^. Standard Age Score (SAS)—A normalized score with a mean of 100 and standard deviation of 16, allowing comparison to same-age peers. Scores range from 50 to 150, with 100 representing average performance for the age group. ^2^. Percentile Rank (P.R.)—Shows the percentage of students in the same age or grade group who scored lower. For example, a percentile of 85 means the student performed better than 85% of peers. Ranges from 1 to 99. ^3^. Stanine (S)—A simplified 9-point scale (1 = lowest, 9 = highest) that groups percentile ranks into broad performance levels (e.g., below average, average, above average). Useful for general classification without fine-grained detail.

**Table 2:** Academic achievement analysis for student M.F.

Subject	Current Performance (%)	3-Semester Avg. (%)	Coefficient of Stability	Expectation (%)	Performance Gap (Actual − Expected)
Mathematics	98	98	0.97	95	+4
Biology	98	97	0.97	93	+5
Physics	100	99	0.95	95	+5
Chemistry	97	96	0.94	90	+7
Computer Science	95	94	0.95	91	+4

**Table 3:** PDAS ratings for student M.F.

**Section 1: Emotional Development**				
Item	1	2	3	4
The student is very sensitive to criticism or feedback.	✓			
The student exhibits a pattern of emotional disturbance (e.g., depression, inappropriate feelings and/or reactions, aggressive behavior, etc.).	✓			
The student reacts aggressively and/or defensively when criticized.	✓			
The student carefully considers feedback and criticism and adjusts her behavior accordingly.				✓
**Section 2: Relationships with Peers**				
The student has very weak interpersonal skills and hardly any friends.	✓			
The student’s interpersonal skills are not as developed as those of her peers.	✓			
The student’s interpersonal skills are appropriate for his age.	✓			
The student demonstrates good interpersonal skills and prefers the company of older children rather than her peers.			✓	
The student has good interpersonal skills with her peers of the same age, as well as with older and younger students.				✓
**Section 3: Student’s Attitude Regarding Full Grade Acceleration**				
The student indicates that she does not want full grade acceleration.				✓
The student is unsure about full grade acceleration.				✓
The student is slightly moderately positive about full grade acceleration.				✓
The student is excited about full grade acceleration.	✓			
**Section 4: Parental Attitude and Support**				
The parents appear to be overly involved in their child’s progress and/or putting pressure on their child.	✓			
The parents appear to be uninterested and uninvolved in their child’s academic progress.	✓			
The parents appear to be generally supportive and involved in their child’s progress.			✓	
The parents are supportive and committed to working with the school to meet their child’s academic needs.				✓

Note: Ratings are based on a 4-point frequency scale: 1 = Rarely/Never, 2 = Sometimes, 3 = Often, and 4 = Always.

**Table 4:** CogAT profile for student S.L.

Battery	Raw Score	Standard Age Score (SAS)	Percentile Rank (PR)	Stanine (S)
**Verbal**	55/65	124	93	8
**Quantitative**	14/60	75	6	2
**Nonverbal**	18/65	75	6	2
**Composite**	87/190	89	25	4

**Table 5:** Academic achievement analysis for student S.L.

Subject	Current Performance (%)	3-Semester Avg. (%)	Coefficient of Stability	Expectation (%)	Performance Gap (Actual − Expected)
**Mathematics**	77	88	0.63	80	−3
**Biology**	97	94	0.91	96	+1
**Physics**	76	88	0.54	78	−2
**Chemistry**	78	82	0.71	82	−4
**Computer Science**	100	97	0.95	99	+1

**Table 6:** PDAS ratings for student S.L.

**Section 1: Emotional Development**				
Item	1	2	3	4
The student is very sensitive to criticism or feedback.		✓		
The student exhibits a pattern of emotional disturbance (e.g., depression, inappropriate feelings and/or reactions, aggressive behavior, etc.).		✓		
The student reacts aggressively and/or defensively when criticized.	✓			
The student carefully considers feedback and criticism and adjusts her behavior accordingly.				✓
**Section 2: Relationships with Peers**				
The student has very weak interpersonal skills and hardly any friends.	✓			
The student’s interpersonal skills are not as developed as those of her peers.	✓			
The student’s interpersonal skills are appropriate for her age.	✓			
The student demonstrates good interpersonal skills and prefers the company of older children rather than her peers.		✓		
The student has good interpersonal skills with her peers of the same age, as well as with older and younger students.		✓		
**Section 3: Student’s Attitude Regarding Full Grade Acceleration**				
The student indicates that she does not want full grade acceleration.	✓			
The student is unsure about full grade acceleration.	✓			
The student is slightly moderately positive about full grade acceleration.	✓			
The student is excited about full grade acceleration.			✓	
**Section 4: Parental Attitude and Support**	✓			
The parents appear to be overly involved in their child’s progress and/or putting pressure on their child.		✓		
The parents appear to be uninterested and uninvolved in their child’s academic progress.		✓		
The parents appear to be generally supportive and involved in their child’s progress.			✓	
The parents are supportive and committed to working with the school to meet their child’s academic needs.			✓	

**Table 7:** CogAT profile for student T.M.

Battery	Raw Score	Standard Age Score (SAS)	Percentile Rank (PR)	Stanine (S)
Verbal	35/65	102	55	4
Quantitative	40/60	103	57	5
Nonverbal	24/65	84	16	3

**Table 8:** Academic achievement analysis for student T.M.

Subject	Current Performance (%)	3-Semester Avg. (%)	Coefficient of Stability	Expectation (%)	Performance Gap (Current − Previous Avg.)
Mathematics	47	84	0.61	61	−37
Biology	89	71	1.06	80	+18
Physics	45	90	0.63	62	−45
Chemistry	90	90	1.00	90	0
Computer Science	90	95	0.95	92	−5

**Table 9:** PDAS ratings for student T.M.

**Section 1: Emotional Development**				
Item	1	2	3	4
The student is very sensitive to criticism or feedback.			✓	
The student exhibits a pattern of emotional disturbance (e.g., depression, inappropriate feelings and/or reactions, aggressive behavior, etc.).		✓		
The student reacts aggressively and/or defensively when criticized.	✓			
The student carefully considers feedback and criticism and adjusts his behavior accordingly.			✓	
**Section 2: Relationships with Peers**				
The student has very weak interpersonal skills and hardly any friends.	✓			
The student’s interpersonal skills are not as developed as those of his peers.	✓			
The student’s interpersonal skills are appropriate for his age.	✓			
The student demonstrates good interpersonal skills and prefers the company of older children rather than his peers.		✓		
The student has good interpersonal skills with his peers of the same age, as well as with older and younger students.		✓		
**Section 3: Student’s Attitude Regarding Full Grade Acceleration**				
The student indicates that he does not want full grade acceleration.	✓			
The student is unsure about full grade acceleration.	✓			
The student is slightly moderately positive about full grade acceleration.			✓	
The student is excited about full grade acceleration.			✓	
**Section 4: Parental Attitude and Support**				
The parents appear to be overly involved in their child’s progress and/or putting pressure on their child.		✓		
The parents appear to be uninterested and uninvolved in their child’s academic progress.		✓		
The parents appear to be generally supportive and involved in their child’s progress.		✓		
The parents are supportive and committed to working with the school to meet their child’s academic needs.			✓	

## Data Availability

The original contributions presented in this study are included in this article. Further inquiries can be directed to the author.
